# Youth’s Experiences of the Devaluing of Their Contributions Due to Their Ethnicity and Gender

**DOI:** 10.1007/s10964-022-01617-1

**Published:** 2022-05-04

**Authors:** Andrew J. Fuligni, Xochitl Arlene Smola, Samir Al Salek

**Affiliations:** University of California, Los Angeles, USA

**Keywords:** Devalued contributions, Discrimination, Marginalization, Young adults, Psychological well being

## Abstract

The message that one’s contributions are devalued can be a significant way that youth experience marginalization during the transition into adulthood. Participants (*N* = 298, *M*_age_ = 19.47 years, 51% female) reported having their ideas, opinions, and contributions being unwelcomed due to their ethnicity and gender. African American, Latinx, and Asian American young women indicated the most frequent devalued contributions. Devalued contributions due to ethnicity and gender were most strongly linked among these groups and Multiethnic youth than European American youth. Devalued contributions predicted depressive symptoms, feeling more needed and useful by society, and a greater sense of purpose beyond a traditional measure of discrimination. Assessing experiences of devalued contributions can provide a more thorough understanding of how marginalization shapes the transition to adulthood.

## Introduction

In addition to creating systematic inequalities in received resources and experienced hostility, marginalization can create disparities in adolescents’ opportunities to make meaningful contributions in their everyday lives. The message that one’s contributions are devalued and unwelcome can be one of the most consequential ways that social marginalization can affect youth (Sumner et al., 2018). Yet, in contrast to the large body of quantitative research on interpersonal discrimination as assessed by experiences of unfair treatment and hostility, inequality in the extent to which youth experience their contributions to the social world as being devalued, unwelcome, and thwarted remains relatively unassessed. This study was designed to examine ethnic and gender differences in the extent to which youth experience the devaluation of their contributions, as well as the implications of such experiences for several aspects of their psychological well being.

Marginalization and inequality operate in multiple forms to shape the adolescent experience, including both structural (e.g., institutional segregation, discriminatory policies, resource inequalities) and interpersonal (e.g., differential treatment, hostility, microaggressions) mechanisms (National Academies of Sciences, 2019). Together, these mechanisms work to marginalize adolescents from groups in the minority and of lower social status in the broader society. Racism is one of the most powerful forms of marginalization in many societies such as the United States (García Coll et al., 1996; Seaton et al., 2018; Spencer, 1995), preventing equitable access to resources and opportunities and creating everyday experiences of unfair treatment, hostility, and othering for those not in the preferred or majority racial and ethnic group (i.e., White, European American). Sexism can generate similar experiences for those who do not identify as being traditionally male (Cole, 2009; Purdie-Vaughns & Eibach, 2008) and both racism and sexism often intersect with adultism, a set of practices and systems that perpetuate the idea that young people are of lesser worth and value as compared to adults (Flasher, 1978). These intersections and the negative experiences that they create for adolescents impact most aspects of development – physical health, psychological well-being, educational success – resulting in disparities according to race, ethnicity and gender (National Academies of Sciences, 2019).

Conceptualizations of marginalization among youth often include the denial and devaluing of one’s contributions due to factors such as race, ethnicity, and gender (Causadias & Umaña-Taylor, 2018; Sumner et al., 2018). Youth may develop a sense of their group being devalued in the larger society by witnessing the structural inequalities and interpersonal hostility faced by other members of their racial, ethnic, and gender group. Such perceptions of the status of one’s larger social group have been assessed in previous studies of African American and Latinx populations (e.g., Cuellar et al., 1995; Sellers et al., 1997). At the same time, adolescents may have direct experiences of their own, individual contributions being denied and unwelcomed in their interpersonal interactions of everyday life. These experiences may include having one’s opinions being ignored or dismissed, not being treated as an asset or valued member of a group, and being excluded from decision-making because of one’s ethnicity, race, or gender (Fuligni, 2020). Interestingly, these experiences have not been typically assessed in research on adolescent development. Studies of interpersonal racial, ethnic, and gender discrimination often focus more on experiences of direct hostility such as being threatened, harassed, or denied service or resources because of one’s race, ethnicity, or gender (Greene et al., 2006; Murry et al., 2001; Williams et al., 1997). This research importantly demonstrates the far-reaching consequences of racial, ethnic or gender-based hostility and unfair treatment for psychological and physical health (Benner et al., 2018). Yet, these studies typically do not assess youth’s direct experiences of having their contributions—their ideas and chances to give support or resources to others – denied, unwelcomed, and devalued by others. A more complete understanding of the impact of marginalization should include the extent to which adolescents experience the denial and devaluation of their contributions in their everyday lives.

Many theoretical models of the fundamental social orientation of human beings emphasize the importance of contributing to the social world by offering ideas, support, and resources to other people and groups (Baumeister & Leary, 1995; Tomasello, 2009). Adolescents have a particular developmental need and desire to contribute to other people, social groups, and the larger society (Fuligni, 2019; Watts et al., 2011). Providing ideas, support, and resources to others assists key developmental tasks such as autonomy and identity. It also has beneficial effects on psychological and physical health through both physiological and psychological mechanisms (Schacter and Margolin, 2018; Schreier et al., 2013). Physiologically, contributing may trigger mechanisms associated with reward and reduced responsivity to stress, threat, and fear (Eisenberger, 2013). Psychologically, contributing to others can confer the sense of value, respect, and status that is particularly salient during the years of adolescence and young adulthood (Yeager et al., 2018).

The understanding of the impact of marginalization on youth can be enhanced by examining group differences in youth’s experiences that their contributions are devalued and unwelcomed. These experiences may be particularly significant during the transition to young adulthood, the developmental period when youth work to establish their role in the larger social world, as this process typically involves consideration of their responsibilities and their desire to have an impact on other people, groups, and society (Arnett, 2000; Burrow et al., 2020; Damon, 2008; Fuligni & Pedersen, 2002). As such, experiences that suggest that one’s contributions are devalued and unwelcomed can present a significant challenge to the transition to young adulthood for youth from marginalized groups. How these experiences may be due to adolescents’ ethnic background and their gender, two of the most significant ways in which adolescents can experience interpersonal discrimination, should be assessed. The intersection of race or ethnicity and gender can be significant for the experiences of young women and those from African American, Latinx, and Asian American backgrounds (Juan et al., 2016). Having one’s opinions, ideas, and contributions denied or dismissed may be particularly acute for women of color as they may encounter intersectional marginalization because of both their gender and ethnicity (Cole, 2009; Purdie-Vaughns & Eibach, 2008). These experiences can be compared to more typically-assessed hostile and unfair everyday discrimination in order to determine whether devalued contributions are an additional aspect of marginalization for youth.

The developmental implications of experiencing devalued contributions for adolescents’ psychological and social adjustment as they make the transition to young adulthood should be assessed. First, as with hostile and unfair discriminatory treatment (Benner et al., 2018), experiencing the denial and devaluing of one’s contributions may be associated with higher levels of depressive symptoms. The physiological and psychological mechanisms associated with contributing and helping—greater reward processing, reduced responsivity to stress and threat, and a sense of respect and value (Eisenberger, 2013)—would be less available to these youth. In addition, the linking devalued contributions with one’s group membership could bring about the same negative impacts upon well-being as found with hostile and unfair discrimination.

Second, experiencing devalued contributions may have implications for one’s sense of purpose and feeling needed and useful by society, two aspects of psychological development closely tied to social integration and a successful transition to adulthood (Damon, 2008; Hill & Burrow, 2021). On the one hand, youth who have their contributions denied may struggle with establishing a sense of purpose and come to believe that they are not needed by the larger society. Such a pathway may be predicted by models of internalized racism and sexism, in which the recipients of chronic levels of such treatment may come to consciously or unconsciously accept and enact the underlying racial and sexual hierarchies (Pyke, 2010). Even without such internalization, the constant thwarting of one’s attempts to contribute to the world may lead adolescents to question their sense of purpose and whether they are needed by their social worlds. On the other hand, marginalization implied by denied contributions could have no impact or actually stimulate purpose, meaning, and a motivation for youth to challenge such dynamics for themselves, their social group, and the broader society (Purdie-Vaughns & Eibach, 2008; Sumner et al. 2018; Watts et al. 2011). Groups experiencing the devaluation of their contributions have developed adaptive cultures and means of action by which such devaluation can be challenged and confronted (Sumner et al., 2018). Indeed, youth from racial, ethnic, and gender groups who traditionally experience devaluation make important contributions to their communities and social worlds in a myriad of ways (Watts & Flanagan, 2007; Wray‐Lake & Abrams, 2020).

## Current Study

The current study was designed to enhance our understanding of the experience of marginalization during adolescence by examining the frequency with which youth experience their contributions being denied and devalued. It was predicted that, consistent with previous research on interpersonal discrimination, those from racial/ethnic minority groups would report more frequent devaluation of their contributions due to ethnicity, and those identifying as female would report greater devaluation due to their gender. To explore the intersectionality of ethnicity and gender and whether ethnic minority women would report the most frequent experiences of devaluation of their contributions, the possibility that experiencing devalued contributions was additionally predicted by the interaction between ethnicity and gender was examined. It was expected that experiencing the devaluing of one’s contributions would predict greater depressive symptoms above-and-beyond the frequency of traditionally-assessed experiences of interpersonal discrimination. Finally, the linkage of experiencing devalued contributions with one’s sense of purpose and feeling needed and useful by the larger society was explored, but there was not a strong hypothesis as to the specific direction of the associations given the reasonable expectations that the associations could be either positive or negative.

## Methods

### Sample

Participants were recruited from the online platform Prolific (prolific.co) that allows individuals to join and complete online research questionnaires if they meet selection criteria established by the researcher. Prolific sample pool members generally are recruited through different social media outlets, online advertising, and word of mouth. In comparison to other online participant recruitment platforms, Prolific provides higher levels of compensation and obtains better quality data with more honest responses and better attention-check performance than other platforms (Peer et al., 2017). Prolific additionally employs multiple processes to prevent bot-like accounts from providing data to studies by restricting signups to only trustworthy IP addresses and common internet service providers, among other processes (Prolific, 2018). The selection criteria for the current study were anyone in the United States from 18 to 21 years of age who self-identified as African American, Hispanic or Latino, or European American. Data collection continued until reaching the targeted sample size of approximately 300 (100 from each ethnic group) in order to provide enough statistical power to detect expected small to medium-sized group differences, correlations, and regression coefficients (Cohen, 1988), with the final total reaching 321 respondents.

Participants took approximately 45 min to complete their questionnaire and were paid $10. Attention-check questions were employed to remove participants who responded randomly and questionnaires that may have been completed by automated programs. These questions included items such as “A dog is to a puppy what a cat is to a _____,” “Please choose ‘Strongly Agree’ for this item,” and “Please write two sentences about your favorite hobby.” Participants who responded inappropriately to any of these questions were removed before analyses (*n* = 22); a participant who reported being 25 years of age was also removed.

The final analytical sample of 298 participants averaged 19.47 (*SD* = 1.12*)* years of age, and 51.34% identified as female and 48.66 as male. Approximately two-thirds (68.12%) of the youth reported being in college at the time of the questionnaire completion, and a small proportion had already received a four-year college degree (4.36%). Although the sample selection criteria did not include Asian American, 17.11% of the youth self-identified as such and were retained for the present analyses. Approximately one-quarter of the sample each identified as European American (20.47%), African American (26.17%), or Hispanic or Latino (26.85%), and the remaining 9.40% of the sample reported multiple ethnicities (labeled here as “Multiethnic”).

Although the pan-ethnic terms of Hispanic or Latino were used in the measurement of ethnicity as these are among the most used by those in the U.S. with Latin American backgrounds (Pew Research Center, 2020), the term Latinx was used in this paper in order to be most gender-inclusive. In addition, although many of those identifying as European American would likely also identify as White and those identifying as African American would likely also identify as Black, the terms “European American” and “African American” are used when referring to results because those are the labels that were assessed in the study. It is unknown how many of the Latinx participants would also identify with the labels “Black” or “White”.

A little more than half of the youth reported being from immigrant families (6.37% first-generation, or foreign-born themselves; 45.64% second-generation, or American-born with at least one foreign-born parent). Almost half (41.95%) of the participants had a mother who graduated from college.

Participants completed the questionnaire from April 30 to May 15, 2020, when school closures and physical distancing rules were put into place in response to the COVID-19 pandemic. A total of 95.56% of those in college were attending school online and 87.92% of the full sample reported living with their parents or other family members. As this was early in the pandemic, actual rates of COVID-19 diagnoses for oneself (1.35%) or someone personally known to the respondent (23.49%) were low.

### Measures

#### Perceived devaluing of contributions

Two measures assessed the frequency with which youth perceived that their contributions were devalued or unwelcome because of their (1) ethnic background and (2) gender. Participants used a 5-point scale that included 1 (“Almost never”), 2 (“Once in a while”), 3 (“Sometimes”), 4 (“Frequently”), 5 (“Almost always”) to respond to 10 items such as “My ideas are not requested because of my ethnic background,” “My contributions are not welcome because of my gender,” “My opinions are ignored because of my ethnic background,” and “My contributions are not valued because of my gender.” The complete measures are presented in Table 1.

**Table 1:** Measures of perceived of devaluation of contributions

1. My ideas are not requested because of my ethnic background [gender].
2. My contributions are not welcome because of my ethnic background [gender].
3. I feel that I am not needed because of my ethnic background [gender].
4. My perspectives are dismissed in group discussions because of my ethnic background [gender].
5. I am not given the chance to offer help or assistance to others because of my ethnic background [gender].
6. I am not viewed as an asset because of my ethnic background [gender].
7. My contributions are not valued because of my ethnic background [gender].
8. I am excluded from group decision-making because of my ethnic background [gender].
9. My opinions are ignored because of my ethnic background [gender].
10. I am not considered useful by others because of my ethnic background [gender].

Separate confirmatory factor analyses (CFAs) for each measure that modeled all items loading onto a single latent factor indicated good model fit (devaluation due to ethnicity: CFI = 0.943, TLI = 0.927, SRMR = 0.036; due to gender: CFI = 0.939, TLI = 0.922, SRMR = 0.029) and item loadings that ranged from 0.67 to 0.93. Multi-group CFAs tested measurement equivalency in the devaluation of contribution due to ethnicity across the different ethnic groups and equivalency in the devaluation due to gender measure across different gender groups. Results indicated good fit for configural, metric, and scalar equivalence in both measures. More details on the CFAs are available in Supplemental Materials. Both measures had strong internal consistency (αs: ethnic = 0.95, gender = 0.97). Mean scores for each measure across all of the constituent items indicated that the frequency of devalued contributions was relatively low (ethnicity: *M* = 1.64, *SD* = 0.82; gender: *M* = 1.63, *SD* = 0.85). In comparison, measures of interpersonal discrimination often obtain levels of internal consistency above 0.90 and similarly low frequencies of occurrence (Greene et al., 2006; Murry et al., 2001).

#### Perceived discrimination

Participants completed a modification of a commonly-used measure of everyday discrimination (Williams et al., 1997, 2008) in which they used a scale that ranged from 1 (“Never”) to 4 (“4 or more times”) to report the frequency with which they experienced unfair treatment (e.g., “You have been treated with less courtesy than other people,” “You have received poorer service than other people at restaurants or stores”), and whether they attributed each experience to their race, gender, age, or height and weight. An indicator of discrimination frequency was created by averaging the frequency responses to all 9 items (*α* = 0.84, *M* = 1.93, *SD* = 0.68). Additional indicators of attributions to race and gender were created by summing the number of experiences that were attributed to each (ethnicity attribution: *M* = 1.84, *SD* = 2.49; gender attribution: *M* = 1.34, *SD* = 1.81).

#### Depressive symptoms

Youth completed the 20-item Center for Epidemiological Studies Depression scale (Radloff, 1977), using a scale that ranged from 1 (“Rarely or none of the time”) to 4 (“Most or all of the time”) to respond to items such as “You felt fearful,” “Your sleep was restless,” and “You felt sad.” The mean was taken of the 20 items of the scale and the measure had a high level of internal consistency (*α* = 0.91, *M* = 2.67, *SD* = 0.66).

#### Sense of meaning and purpose

Participants used a 1 (“Almost never”) to 5 (“Almost always”) scale to complete a 5-item measure of the presence of youth’s sense of meaning and purpose (Steger et al., 2006). Items included “I understand my life’s meaning,” “My life has a clear sense of purpose,” and “I have discovered a satisfying life purpose.” The mean was taken of the five items and the measure had a high level of internal consistency (*α* = 0.92; *M* = 2.73, *SD* = 1.11).

#### Feeling needed and useful in society

Youth completed a measure assessing their sense of being needed and useful in society. Conceptually, a key psychological mediator of being able to contribute to the social world is the sense that one is needed and useful, which has been linked to psychological well-being and is a common theme in adolescents’ narratives about their desires to have an impact upon the world (Burrow et al., 2020; Damon, 2008). The measure was included in order to examine whether perceiving that one’s contributions are devalued due to ethnicity or gender compromised this important sense of being needed and useful to society at the transition to adulthood. The measure was based upon similar measures that assessed feeling needed and useful by family and friends (psychometric information available in Fuligni et al., 2021). Participants used a scale that ranged from 1 (“Almost never”) to 5 (“Almost always”) to respond to five questions: “I feel useful in society,” “I feel needed in society,” “I feel that society can depend upon me,” “I feel that I contribute to society,” and “I feel that I am a good member of society.” The mean was taken of the five items and the measure had a high level of internal consistency (*α* = 0.92; *M* = 2.60, *SD* = 1.06).

### Analysis Plan

First, ethnic and gender differences in adolescents’ experiences of devaluation of their contributions and discrimination were examined using ethnic x gender analyses of covariance (ANCOVA) with parental education and generational status treated as covariates. Significant effects were followed up with Bonferroni contrasts of group differences, as appropriate. Bivariate correlations estimated the associations between the different measures of devalued contributions and discrimination, as well as the associations of devalued contributions with depressive symptoms, sense of meaning and purpose, and feeling needed and useful by society. Finally, ordinary least squares hierarchical regression models examined whether associations of devalued contributions with well-being were significant above-and-beyond discrimination.

## Results

### Experiences of Devalued Contributions

Mean levels of youth’s experiences with having their contributions being devalued due to their ethnicity and gender are presented in Table 2. An ethnicity x gender ANCOVA with parental education (mean-centered) and generational status (first vs. third, second vs. third) as covariates suggested that although the experience was relatively infrequent (i.e., between “1 = almost never” to “2 = once in a while”), youth from African American and Asian American backgrounds perceived more devaluation of their contributions due to their ethnicity than did European American youth, and that African American youth reported more devaluation than did Latinx youth, *F*(4,277) = 6.78, *p* < 0.001, Bonferroni contrasts, *p* < 0.05. Female youth reported more devaluation due to ethnicity than did their male counterparts, *F*(1,277) = 5.76, *p* = 0.017. These main effects, however, were moderated by a significant ethnicity x gender interaction (*F*(4,277) = 2.76, *p* = 0.028) by which female African American, Latinx, and Asian American youth reported greater devaluation of their contributions due to their ethnicity than male members of the same ethnic group, *p*s = 0.002–0.031. The gender difference was not significant among European American and Other/Multiethnic youth, *p*s = 0.561, 0.126. There were no differences according to parental education and generational status, *F*s(1,277) = 0.00–0.21, *p*s = 0.651–0.995.

**Table 2:** Ethnic and gender differences in perceived devalued contributions due to ethnicity and gender

	Due to ethnicity		Due to gender	
Participant ethnicity & gender	M	SD	M	SD
*European American*	1.25	0.50	1.55	0.74
Female	1.32	0.63	1.86	0.88
Male	1.20	0.36	1.30	0.50
*African American*	1.90	0.97	1.68	0.97
Female	2.19	1.11	2.03	1.08
Male	1.60	0.70	1.31	0.66
*Latinx*	1.55	0.75	1.61	0.84
Female	1.80	0.86	2.08	0.89
Male	1.29	0.49	1.30	0.72
*Asian American*	1.81	0.83	1.70	0.86
Female	2.02	0.86	2.08	0.89
Male	1.55	0.73	1.24	0.54
*Multiethnic*	1.68	0.82	1.61	0.75
Female	1.53	0.62	1.74	0.88
Male	1.92	1.04	1.41	0.46
*Total*	1.64	0.82	1.63	0.85
Female	1.83	0.92	1.94	0.93
Male	1.44	0.65	1.30	0.61

The same ANCOVA indicated that female youth reported a greater devaluation of their contributions due to their gender than male youth, *F*(1,277) = 35.08, *p* < 0.001. There were no differences according to parental education, and generational status, *F*s(1,277) = 0.23–2.24, *ps* = 0.136–0.634, and neither ethnicity and nor the interaction between ethnicity and gender were significant, *F*s(4,277) = 0.20, 0.71, *ps* = 0.939, 0.585.

The measures of perceived devaluation of contribution due to ethnicity and gender were significantly correlated at *r* = 0.56, *p* < 0.001. An analysis of covariance (ANCOVA) with tests of equal slopes indicated that the association between the two measures significantly differed according to ethnicity, *F*(4,288) = 4.83, *p* = 0.001. Simple slopes analyses of each ethnic group indicated that the association between the two measures of devalued contributions was significant for African American, Asian American, Latinx, and Multiethnic youth (*b*s = 0.67, 0.62, 0.52, 0.43, respectively, *p*s < 0.05–0.001) but not among European American youth (*b* = 0.11, *p* = 0.21).

### Discrimination

As shown in Table 3, bivariate correlations indicated that youth’s perceptions of the devaluation of their contributions because of their ethnicity and gender were moderately associated with reported discrimination.

**Table 3:** Correlations of perceived devalued contributions and perceived discrimination and attributions

	Contribution devaluation	
	Due to ethnicity	Due to gender
Discrimination	*r*	*r*
Frequency	0.34^***^	0.36^***^
Race Attribution	0.45^***^	0.39^***^
Gender Attribution	0.39^***^	0.46^***^

Note: *p < 0.05, **p < 0.01, ***p < 0.001

Mean levels of youth’s reports of discrimination and their attributions are presented in Table 4. An ethnicity x gender ANCOVA with parental education and generational status as covariates suggested that participants from African American backgrounds reported more frequent discrimination than their European American peers, F(4277) = 3.62, *p* = 0.007, Bonferroni contrast, *p* < 0.05. There were no differences according to gender, parental education, and generational status, *F*s(1277) = 0.46–2.04, *ps* = 0.154–0.497, and the interaction between ethnicity and gender was not significant, *F*(4277) = 0.44, *p* = 0.777.

**Table 4:** Ethnic and gender differences in perceived discrimination

	Discrimination frequency		Discrimination race attribution		Discrimination gender attribution	
Participant ethnicity & gender	M	SD	M	SD	M	SD
*European American*	1.68	0.55	0.15	0.44	1.34	1.91
Female	1.75	0.55	0.19	0.56	2.22	2.19
Male	1.62	0.56	0.12	0.33	0.65	1.30
*African American*	2.09	0.84	3.19	3.02	1.17	1.73
Female	2.22	0.86	3.55	3.12	1.52	1.71
Male	1.95	0.81	2.82	2.90	0.79	1.70
*Latinx*	2.00	0.59	1.53	1.97	1.56	1.99
Female	2.13	0.64	1.54	2.13	2.24	2.08
Male	1.87	0.51	1.51	1.80	0.85	1.61
*Asian American*	1.81	0.59	2.18	2.28	1.02	1.58
Female	1.83	0.57	2.14	1.99	1.54	1.64
Male	1.79	0.63	2.22	2.63	0.39	1.27
*Multiethnic*	2.02	0.73	2.07	2.92	1.79	1.66
Female	2.03	0.64	1.47	2.55	2.47	1.74
Male	2.00	0.89	3.00	3.32	0.73	0.79
*Total*	1.93	0.68	1.84	2.49	1.34	1.81
Female	2.02	0.69	1.93	2.54	1.95	1.91
Male	1.83	0.66	1.75	2.44	0.70	1.46

The same ANCOVA was repeated for discrimination attributions, suggesting that African American, Asian American, and Multiethnic youth reported more ethnicity-based attributions than their European American peers, and that African American youth reported more than did Latinx and Asian American youth, *F*(4,277) = 16.51, *p* < 0.001, Bonferroni contrasts, *p* < 0.05. There were no differences in ethnicity-based attributions according to gender, parental education, or generational status *F*s(1,277) = 0.66–1.79, *ps* = 0.182–0.418, nor was the interaction between ethnicity and gender statistically significant, *F*(4,277) = 1.38, *p* = 0.243.

Female youth reported significantly more gender-based attributions (*M* = 1.95*, SD* = 1.91) than did males, *M* = 0.70*, SD* = 1.46, *F*(1,277) = 33.94, *p* < 0.001. There were no differences in gender-based attributions according to parental education or generational status *F*s(1,277) = 0.00–0.57, *ps* = 0.450–0.962, and neither ethnicity nor the interaction between ethnicity and gender were significant predictors, *F*s(4,277) = 1.42, 0.70, *ps* = 0.228, 0.592.

### Depressive Symptoms, Meaning, and Feeling Needed and Useful in Society

Bivariate correlations suggested that youth’s perceptions of their contributions being devalued because of their ethnicity and gender were associated with greater depressive symptoms (ethnicity: *r* = 0.27, gender: *r* = 0.34, *p*s < 0.001), but not youth’s sense of meaning and purpose (ethnicity: *r* = 0.06, gender: *r* = 0.01, *p*s = 0.301, 0.811) or feeling needed and useful in society (ethnicity: *r* = 0.09, gender: *r* = 0.01, *p*s = 0.106, 0.887).

Hierarchical ordinary least squares regression models predicted psychological well being from perceived devalued contributions due to ethnicity or gender both before (Step 1) and after controlling for the frequency and attribution of discrimination due to race or gender (Step 2). Ethnicity was dummy-coded with European American as the baseline in these models for statistical reasons, not to imply that this group represents the norm to which all other groups should be compared. As reported above, youth from European American backgrounds reported the lowest frequency of devalued contributions as compared to other ethnic groups, and analyses were designed to account for those particular associations with ethnicity when estimating the association of devalued contributions with the depressive symptoms. Gender was coded 1 = female, 0 = male. Additional controls included mean-centered parental education, nativity (first and second-generation vs. third), and college attendance (no college = 1, attending or completed college = 0).

As shown in Tables 5 and 6, youth’s experiences with their contributions being devalued due to ethnicity or gender predicted greater depressive symptoms. The significant associations between devalued contributions due to ethnicity or gender and depressive symptoms remained significant even after including discrimination frequency and attributions in the models. In terms of control variables, African American youth reported fewer symptoms and female youth reported greater symptoms of depression when compared to the baseline group of European American, third generation youth. Second-generation youth evidenced fewer symptoms of depression in the model of ethnic devaluation of contribution, but not the model of gender devaluation.

**Table 5:** Predicting psychological well being from devalued contributions due to ethnicity

	Depressive symptoms				Meaning & purpose				Needed & useful in society			
	*B* (SE)	*β*	*B* (SE)	*β*	*B* (SE)	*β*	*B* (SE)	*β*	*B* (SE)	*β*	*B* (SE)	*β*
Female	0.15 (0.04)^***^	0.23	0.13 (0.04)^***^	0.19	−0.04 (0.07)	−0.04	−0.03 (0.07)	−0.03	−0.11 (0.06)	−0.10	−0.09 (0.06)	−0.08
African American	−0.26 (0.11)^*^	−0.17	−0.27 (0.11)^*^	−0.18	−0.15 (0.20)	−0.06	−0.07 (0.21)	−0.03	−0.20 (0.18)	−0.08	−0.23 (0.19)	−0.10
Latinx	−0.07 (0.12)	−0.05	0.01 (0.11)	0.01	−0.08 (0.21)	−0.03	−0.02 (0.21)	−0.01	−0.45 (0.19)^*^	−0.19	−0.43 (0.19)^*^	−0.18
Asian American	−0.07 (0.13)	−0.04	0.13 (0.12)	0.08	−0.27 (0.24)	−0.09	−0.29 (0.24)	−0.10	−0.74 (0.22)^***^	−0.27	−0.80 (0.22)^***^	−0.29
Multiethnic	−0.03 (0.15)	−0.01	−0.07 (0.14)	−0.03	−0.36 (0.26)	−0.10	−0.30 (0.26)	−0.08	−0.37 (0.24)	−0.10	−0.38 (0.24)	−0.10
Parental education	0.00 (0.02)	0.01	0.00 (02)	0.02	0.03 (0.03)	0.06	0.03 (0.03)	0.05	0.07 (0.03)^**^	0.16	0.07 (0.03)^**^	0.16
First generation	−0.18 (0.17)	−0.06	−0.23 (16)	−0.08	0.19 (0.31)	0.04	0.24 (0.30)	0.05	−0.01 (0.28)	−0.00	0.01 (0.28)	0.00
Second generation	−0.16 (0.09)	−0.12	−0.18 (08)^*^	−0.13	0.25 (0.16)	0.11	0.28 (0.16)	0.13	0.39 (0.15)^**^	0.19	0.39 (0.15)^**^	0.19
No College	0.14 (0.08)	0.10	0.12 (07)	0.09	−0.20 (0.14)	−0.09	−0.19 (0.14)	−0.08	−0.25 (0.13)	−0.11	−0.23 (0.13)	−0.11
Devalued contributions	0.21 (0.05)^***^	0.26	0.14 (0.05)^**^	0.18	0.11 (0.09)	0.08	0.20 (0.09)^*^	0.15	0.17 (0.08)^*^	0.14	0.19 (0.10)^*^	0.15
Discrim – Frequency			0.58 (0.08)^***^	0.44			−0.44 (0.16)^**^	−0.20			−0.31 (0.15)^*^	−0.15
Discrim – Race			−0.03 (0.02)	−0.12			−0.01 (0.04)	−0.01			0.04 (0.03)	0.09
*R*^2^		0.16^***^		0.30^***^		0.03		0.07		0.11^***^		0.12^***^

Note: Ethnicity, generational status, and college status were dummy-coded with European American, third generation, and whether participants completed any amount of college as the baseline.

*p < 0.05, **p < 0.01, ***p < 0.001

**Table 6:** Predicting psychological well being from devalued contributions due to gender

	Depressive symptoms				Meaning & purpose				Needed & useful in society			
	*B* (SE)	*β*	*B* (SE)	*β*	*B* (SE)	*β*	B (SE)	*β*	*B* (SE)	*β*	B (SE)	*β*
Female	0.12 (0.04)^**^	0.04	0.12 (0.04)^**^	0.18	−0.04 (0.07)	−0.03	−0.06 (0.07)	−0.06	−0.09 (0.07)	−0.09	−0.10 (0.07)	−0.10
African American	−0.14 (0.11)	0.11	−0.26 (0.10)^*^	−0.17	−0.08 (0.19)	−0.03	0.07 (0.20)	0.03	−0.09 (0.18)	−0.04	−0.03 (0.18)	−0.01
Latinx	0.11 (0.11)	0.11	−0.02 (0.11)	0.01	−0.06 (0.21)	−0.02	0.03 (0.21)	0.01	−0.41 (0.19)^*^	−0.17	−0.37 (0.19)	−0.16
Asian American	0.13 (0.13)	0.13	0.14 (0.12)	0.08	−0.23 (0.24)	−0.08	−0.18 (0.24)	−0.06	−0.67 (0.22)^**^	−0.24	−0.65 (0.22)^**^	−0.24
Multiethnic	0.05 (0.14)	0.14	−0.05 (0.13)	−0.02	−0.32 (0.26)	−0.08	−0.24 (0.26)	−0.06	−0.30 (0.24)	−0.08	−0.27 (0.24)	−0.07
Parental education	0.01 (0.02)	0.02	0.01 (0.02)	0.02	0.03 (0.03)	0.06	0.03 (0.03)	0.05	0.07 (0.03)^**^	0.16	0.07 (0.03)^*^	0.16
First generation	−0.18 (0.17)	0.17	−0.23 (0.16)	−0.08	0.20 (0.31)	0.04	0.26 (0.30)	0.06	0.00 (0.28)	0.00	0.03 (0.28)	0.01
Second generation	−0.11 (0.09)	0.09	−0.16 (0.08)	−0.12	0.27 (0.16)	0.12	0.30 (0.16)	0.13	0.41 (0.15)^**^	0.20	0.42 (0.15)^**^	0.20
No college	0.12 (0.08)	0.08	0.13 (0.07)	0.09	−0.21 (0.14)	−0.09	−0.20 (0.14)	−0.09	−0.27 (0.13)^*^	−0.12	−0.27 (0.13)^*^	−0.12
Devalued contributions	0.22 (0.05)^***^	0.05	0.11 (0.05)^*^	0.15	0.03 (0.08)	0.03	0.08 (0.09)	0.06	0.06 (0.08)	0.05	0.08 (0.09)	0.07
Discrim – Frequency			0.49 (0.08)^***^	0.36			−0.52 (0.15)^***^	−0.23			−0.22 (0.14)	−0.10
Discrim – Race			0.01 (0.02)	0.03			0.07 (0.04)	0.12			0.03 (0.04)	0.05
*R*^2^		0.17^***^		0.29^***^		0.03		0.07		0.09^**^		0.10^**^

Note: Ethnicity, generational status, and college status were dummy-coded with European American, third generation, and whether participants completed any amount of college as the baseline

*p < 0.05, **p < 0.01, ***p < 0.001

Experiencing devalued contributions due to ethnicity was associated with feeling more needed and useful both before and after controlling for discrimination and with a greater sense of meaning and purpose after controlling for discrimination (see Table 5). Devalued contributions due to gender were unassociated with feeling needed and useful and a sense of meaning and purpose. Discrimination frequency was associated with feeling less needed and useful and a lower sense of meaning and purpose. In terms of control variables, Latinx and Asian American youth reported feeling less needed and useful by society, whereas second-generation and those whose parents had higher levels of education felt more needed and useful.

## Discussion

The message that one’s contributions are devalued and unwelcome can be a significant way in which youth of color and young women experience marginalization as they are working to find their place in the broader social world during the transition to young adulthood. Findings suggest that these messages may be more common for young women of color who reported the greatest frequency of experiencing devalued contributions, when considering both those due to their ethnicity and those due to their gender. Additionally, these two types of experiences were more strongly correlated among youth from African American, Latinx, Asian American, and Multiethnic backgrounds, as opposed to those from European American backgrounds. Finally, although experiencing devalued contributions predicted greater depressive symptoms beyond hostile and unfair discriminatory treatment, other results highlighted how youth’s sense of purpose and being of use to society were positively associated with experiencing devalued contributions. Together, these findings highlight the importance of assessing experiencing one’s contributions as unwelcomed or devalued in order to obtain a better understanding of the ways that marginalization shapes the experiences of youth as they make the transition to young adulthood.

Racism, sexism, and ageism interact to shape experiences of marginalization for many youth in ethnic and racial minority groups or of lower social status in the broader society (Flasher, 1978; Purdie-Vaughns & Eibach, 2008; Seaton et al., 2018). This intersection was particularly evident in the in the experiences of devalued contributions among young women of color. First, the perceived frequency of devalued contributions due to ethnicity was highest among African American, Latinx, and Asian American young women, in addition to the high levels of devalued contributions due to gender that they and women from other ethnic backgrounds reported. Second, there was a greater association between devaluation due to ethnicity and gender among ethnic minority youth as compared to those identifying as European American. Together, these findings are consistent with other work suggesting that young women of color see a greater link between their race or ethnicity and gender than do young White women (Juan et al., 2016). The results highlight the intersection of gender and ethnicity in shaping the types of marginalization experiences of young adults (Cole, 2009; Purdie-Vaughns & Eibach, 2008). The fact that the interaction of gender and ethnicity predicted devalued contributions due to ethnicity, but not discrimination frequency or attribution to race, suggests that examining devalued contributions would provide unique information about the intersectionality of these two social categories in shaping the experiences of marginalization among young adults. Future work should consider adding the assessment of devalued contributions to other commonly-used measures of interpersonal and everyday discrimination.

Very few young adults identifying as European American or male reported experiences of devaluation due to ethnicity or gender, suggesting that this experience was largely absent from their attempts to offer their ideas, perspectives, and contributions in their everyday lives. The lack of experiencing devalued contributions represents an example of how their privileges in American society (Helms, 2017), which they typically do not recognize as such (Brown et al., 2019), become enacted in everyday life as they make the transition to adulthood. Why a small number of European American or male youth reported a devaluing of their contributions due to their ethnic background and gender, such as whether they believed they were experiencing “reverse” racism and sexism, is unclear from the present study and should be examined in future research.

Adolescents and young adults have a developmental need to contribute by providing support, resources, and their ideas to their social worlds, and the denial of such opportunities could have implications for their adjustment during this period of transition (Fuligni, 2020). Findings suggested that experiencing unwelcomed or devalued contributions uniquely predicted multiple aspects of psychological well being above-and-beyond traditionally assessed hostile and discriminatory treatment. First, more frequent experiences of devaluation predicted higher levels of depressive feelings. Denying youth the opportunity to contribute can prevent them from benefitting from the physiological and psychological mechanisms that confer better psychological well-being, including feelings of reward, reduced stress-responsivity, and sense of respect and status (Eisenberger, 2013; Yeager et al., 2018). As with the hostile treatment of interpersonal, everyday discrimination that is typically associated with depressive symptoms (Benner et al., 2018), having one’s opportunity to contribute to the social world thwarted and devalued potentially takes a toll on one’s psychological well-being.

In contrast to what was observed for depressive symptoms, youth’s sense of purpose and being needed and useful to society were positively predicted by having their contributions unwelcomed and devalued due to their ethnicity within the regression analyses. Previous research has demonstrated how youth from oppressed and marginalized groups consistently make important contributions to their social word, whether at home (Hernández & Bámaca‐Colbert, 2016) or in their communities (Watts & Flanagan, 2007), particularly during the transition to adulthood (Wray-Lake et al., 2020). For many youth, marginalization can serve as a fuel and motivator for actively working to make a difference in the world (Sumner et al., 2018; Wray‐Lake & Abrams, 2020). It is unknown what resources or sources of support were available to participants in this study to maintain their sense of purpose and being needed and useful to society. Future work should include supports and opportunities provided by families, peers, and communities that may help young adults use those experiences of devaluation to stimulate a greater sense of purpose and feeling of use to the larger society (Burrow et al., 2020).

The regression models revealed several group and individual differences in psychological well being. African American youth reported fewer depressive symptoms, as has sometimes been observed in previous studies, and females reported greater symptoms consistent with most research (Anderson & Mayes, 2010). Latinx and Asian American youth reported feeling less needed and useful by society, whereas second-generation youth and those whose parents had higher levels of education felt more needed and useful. The ethnic differences in feeling needed and useful may reflect aspects of marginalization experienced by Latinx and Asian American youth not captured in this study, such as greater experiences of microaggressions targed toward these groups (Huynh, 2012) or being seen as a “perpetual foreigner” (Huynh et al., 2011). In contrast, those whose parents obtained higher levels of education may have more optimistic views of their occupational and economic futures, which in turn may translate into a greater impact on the social world (Boehm et al., 2015). Second-generation youth feeling more needed and useful by society may reflect the high aspirations often associated with having immigrant parents (Perreira et al., 2006). Why the same finding was not evident for first-generation youth is more difficult to explain and more research will need to determine the replicability of the generational differences observed in this study.

The findings should be considered in light of several limitations that can be addressed in future research of the devaluation of youth’s contributions. Ethnic, racial, and gender identification should be addressed in a more comprehensive manner. Notably, participants were not asked about their identification with racial categories (e.g., White, Black) and the measure of devalued contributions due to ethnicity did not include reference to race. These absences prevented the inclusion of these critical social categories play a role in marginalization in American society (Richeson & Sommers, 2016). Discrimination measures typically include reference to “race,” either by itself or in combination with “ethnicity” and “ethnic background” (Greene et al., 2006; Murry et al., 2001; Williams et al., 1997). The ethnic differences and prediction of depressive symptoms observed in this study were consistent with those found in studies of racial discrimination, but future uses of the measure of devalued contributions could include reference to race in order to better capture the breadth of this particular experience of marginalization. Additionally, probing racial and ethnic identity more deeply with established scales (e.g., Sellers et al., 1997) would provide insight into how experiences of devalued contributions interact with ongoing racial and ethnic identity development to shape the transition to adulthood. Gender identification was assessed using only the binary option of male or female, preventing many youth from expressing their own unique identification that may additionally intersect with ethnicity to shape experiences of marginalization and discrimination (Shields, 2008). In addition, not measuring other positionalities such as sexual orientation, family income, or disability status as additional contributors to experiencing devalued contributions was a limitation that should be addressed in future studies.

Addressing other limitations would expand the scope and understanding of the breadth of experiencing devalued contributions across the period of adolescence and young adulthood. Devalued contributions were assessed generally and without regard to specific context (e.g., school, work). Such specification could be added to the measures in order to examine more precisely where youth more frequently experience such devaluation and whether some settings may compensate for such experiences by providing more opportunities to contribute. Including participants younger than 18 years of age and a longitudinal design would provide a better picture of how experiencing devalued contributions changes across adolescence and the transition to adulthood, and would allow for untangling the potential bidirectional effects with psychological well-being. Finally, it is unknown how the significant events of 2020, including both the emergence of the COVID-19 pandemic and the racial justice demonstrations in the United States, may have influenced the results. Future studies can explore potential differences according to the timing of data collection and personal experiences with these events.

## Conclusion

The transition to young adulthood is a critical time of life for youth to figure out their role in the larger social world by considering their responsibilities and desire to have an impact on other people, groups, and society. As such, a more complete understanding of the role of marginalization during the transition to adulthood should add systematic examinations of having one’s contributions devalued and unwelcomed. Such work could identify the unique ways that race, ethnicity, and gender intersect to shape experiences of devalued contributions, creating potentially unique challenges for young women of color. At a period of life when establishing one’s place and role in the social world is a critical developmental task, the message that one cannot or should not contribute could be one of the most significant ways that marginalization affects the psychosocial development of marginalized youth. At the same time, given that many of these youth actively work to confront systems of marginalization and inequality, future studies could identify how being unfairly confronted with experiences of devalued contributions may stimulate youth to establish a sense of meaning, purpose, and place in society during the critical transition into adulthood.

## Supplementary information

Supplementary Information
