# Supplementary material for: Youth’s Experiences of the Devaluing of Their Contributions Due to Their Ethnicity and Gender
Source: J Youth Adolesc. 2022 May 4;51(8):1667–78. doi: 10.1007/s10964-022-01617-1 (PMC9067546; doi:10.1007/s10964-022-01617-1)
Supplement: Supplementary file 1 — Supplementary Information [file 10964_2022_1617_MOESM1_ESM.docx]

| Table S1. Confirmatory Factor Analysis Loadings | | |
| --- | --- | --- |
|  | Devalued Contributions | |
|  | Unstandardized Loadings (SE) | Standardized Loadings |
| Ethnicity |  |  |
| Item 1 | 1.00 (–) | 0.67 |
| Item 2 | 1.08 (0.09) | 0.76 |
| Item 3 | 1.04 (0.09) | 0.72 |
| Item 4 | 1.31 (0.10) | 0.84 |
| Item 5 | 1.18 (0.09) | 0.80 |
| Item 6 | 1.23 (0.10) | 0.76 |
| Item 7 | 1.35 (0.10) | 0.90 |
| Item 8 | 1.33 (0.10) | 0.88 |
| Item 9 | 1.42 (0.10) | 0.87 |
| Item 10 | 1.33 (0.10) | 0.84 |
| Gender |  |  |
| Item 1 | 1.00 (–) | 0.81 |
| Item 2 | 1.03 (0.05) | 0.89 |
| Item 3 | 0.96 (0.06) | 0.82 |
| Item 4 | 1.08 (0.05) | 0.91 |
| Item 5 | 1.01 (0.06) | 0.81 |
| Item 6 | 1.12 (0.06) | 0.92 |
| Item 7 | 1.18 (0.06) | 0.93 |
| Item 8 | 0.97 (0.06) | 0.83 |
| Item 9 | 1.06 (0.06) | 0.86 |
| Item 10 | 1.12 (0.06) | 0.88 |
| *Note.* Factor loadings obtained from CFAs on the entire sample with a single latent factor, as described in the Measures section. | | |

Testing Measurement Invariance

We tested the invariance of the parameters of the item factor model for the devalued contribution measure between genders (male and female) and between ethnic groups (European American, African American, Latinx, and Asian American). The *Multiethnic* ethnic category was not included in these analyses as the sample size was quite small (n=28; Kim et al., 2014). We analyzed three increasingly stringent models of factorial invariance: (a) configural invariance, which implies equivalent factor structures between groups; (b) metric or weak invariance, which implies equivalence of loadings (λs) across groups; and (c) scalar or strong invariance, which implies equivalence of loadings and item thresholds (τs).

Given the naturally low-endorsed nature of our measure, we were unable to use traditional approaches. In essence, the lack of endorsement or variability to specific items by a group was problematic for most SEM software. To circumvent the need to have the same response data measured in each group, measurement invariance was tested considering the recommendation of Widaman and colleagues (2013) for using the mixture modeling framework for specification and estimation while treating class membership as known (i.e., Pattern Mixture Procedure with Known Groups). This approach was conducted using *Mplus* (Muthén & Muthén, 2007) which does not provide absolute and global indexes of model fit (e.g., CFI, RMSEA). We instead compared the post-hoc fit of the models using the likelihood ratio test (-2LL) and the Bayesian Informatics Criteria (BIC).

*Invariance across ethnic groups.* The comparison of configural (-2LL=3,921.06, BIC =5,057.54) and metric (-2LL=3,949.55, BIC = 4,934.88) models was nonsignificant (Δ χ^2^ (27) = 28.49, *p* = .386) indicating that the fit of metric invariance was not significantly different from the configural model. With the decreasing BIC, these results suggest that the unifactorial structure of devalued contributions applied to the ethnic groups equally and that the item factor loadings were not significantly different.

The fits of the metric and scalar models (-2LL=4,068.43, BIC = 4,398.74) were not significantly different (Δ χ^2^ (117) = 118.88, *p* = .434) indicating that the fit of scalar invariance was not significantly worse than the metric model. The decreasing BIC confirmed this conclusion. These results suggest that the item loadings and item thresholds were similar across ethnic groups. Thus, the devalued contribution measure is tapping into the same construct in the same scale across different ethnic groups.

*Invariance across gender.* The fit of the configural (-2LL=3,561.04, BIC =4,126.48) and metric (-2LL=3,576.38, BIC = 4,088.42) invariance models was nonsignificant (Δ χ^2^ (9) = 15.34, *p* = .082) indicating that the fit of metric invariance was not significantly worse than the fit of the configural model. Additionally, the BIC favored the metric invariance model.

The scalar invariance model (-2LL=3,637.18, BIC = 3,933.89) fit significantly worse than the metric invariance model (Δ χ^2^ (39) = 60.8, *p* = .014), indicating the item thresholds, as a group, were significantly different across genders. However, the BIC favored the scalar invariance model (Δ BIC = 536.14) suggesting the thresholds were not practically different across genders. Taken together, the scalar invariance model for gender was considered satisfactory.

References

Kim, S. Y., Mun, E. Y., & Smith, S. (2014). Using mixture models with known class membership to address incomplete covariance structures in multiple‐group growth models. *British Journal of Mathematical and Statistical Psychology*, *67*(1), 94-116.

Muthén, L. K., & Muthén, B. O. (2007). *Mplus user’s guide* (5^th^ ed.). Los Angeles, CA: Muthén & Muthén.

Widaman, K. F., Grimm, K. J., Early, D. R., Robins, R. W., & Conger, R. D. (2013). Investigating factorial invariance of latent variables across populations when manifest variables are missing completely. *Structural equation modeling: a multidisciplinary journal*, *20*(3), 384-408
